# Personal

**Published:** 1911-03

**Authors:** 


### PERSONAL

Dr. Lawrence Hendee, of Buffalo, gave a dinner February 7, in honor of Dr. J. M. T. Finney, of Baltimore, who addressed the Buffalo Academy of Medicine that same evening.

Dr. Charles T. Crance, a graduate of the University of Buffalo, class of 1900, has been reappointed health officer of North Tonawanda, N. Y., to serve during 1911.

Dr. Harvey W. Wiley, of Washington, D. C., chief of the Bureau of Chemistry of the Agricultural Department and the most famous pure food expert in this country, delivered an address at the Central Y. M. C. A., Buffalo, on the evening of January 31. Dr. Wiley's topic was, The Public Health—Our Greatest National Asset. The address was delivered under the auspices of the Buffalo Academy of Medicine, Erie County Medical Society, Western New York Section, American Chemical Society and the Erie County Pharmaceutical Association.

Drs. A. E. Woehnert, James E. King and L. Kaufman, of Buffalo, read papers before the Cattaraugus County Medical Society at the meeting held at Salamanca on January 3.

Dr. Horace M. Edmonds, of Tonawanda, N. Y., has been appointed health officer of that city for the year 1911.

Dr. John B. Deaver, of Philadelphia, has been appointed professor of clinical surgery in the University of Pennsylvania, to succeed Dr. Edward Martin, who becomes John Rea professor of dermatology.

Dr. George E. Ellis, of Dunkirk, N. Y., a graduate of the medical department of the University of Buffalo (1891), has been reappointed health officer of that city.

Dr. John D. Howland has been appointed medical superintendent of the Erie County Hospital, the appointment taking effect March 1, 1911. He resigned as deputy medical examiner, an office he had held for several years in order to accept of his new position.

